# Association of stress induced hyperglycemia with angiographic findings and clinical outcomes in patients with ST-elevation myocardial infarction

**DOI:** 10.1186/s12933-022-01578-6

**Published:** 2022-07-26

**Authors:** Nikolaos Stalikas, Andreas S. Papazoglou, Efstratios Karagiannidis, Eleftherios Panteris, Dimitrios Moysidis, Stylianos Daios, Vasileios Anastasiou, Vasiliki Patsiou, Triantafyllia Koletsa, George Sofidis, Georgios Sianos, George Giannakoulas

**Affiliations:** 1 First Department of Cardiology, AHEPA University Hospital, Aristotle University of Thessaloniki, St. Kiriakidi 1, 54636 Thessaloniki, Greece; 2 Laboratory of Forensic Medicine and Toxicology, School of Medicine, Aristotle University of Thessaloniki, 54124 Thessaloniki, Greece; 3 Biomic_Auth, Bioanalysis and Omics Lab, Centre for Interdisciplinary Research of Aristotle University of Thessaloniki, Innovation Area of Thessaloniki, 57001 Thermi, Greece; 4 Pathology Department, Faculty of Medicine, Aristotle University of Thessaloniki, Thessaloniki, Greece

**Keywords:** STEMI, Stress induced hyperglycemia, Diabetes, Thrombus, Distal embolization

## Abstract

**Background:**

Stress induced hyperglycemia (SIH) is common among patients with ST-elevation myocardial infarction (STEMI), even in patients without diabetes mellitus. However, evidence regarding its role on the angiographic outcomes and the prognosis of patients presenting with STEMI is scarce.

**Methods:**

This study included 309 consecutively enrolled STEMI patients undergoing primary percutaneous coronary intervention (pPCI). Patients were diagnosed with SIH if blood glucose on admission was > 140 mg/dl. Also, patients had to fast for at least 8 hours before blood sampling. The objective was to assess whether SIH was associated with major adverse cardiovascular and cerebrovascular (MACCE) events and explore its relationship with angiographic predictors of worse prognosis such as poor initial TIMI flow, intracoronary thrombus burden, distal embolization, and presence of residual thrombus after pPCI.

**Results:**

SIH in diabetic and non-diabetic patients was associated with a higher incidence of LTB (aOR = 2.171, 95% CI 1.27–3.71), distal embolization (aOR = 2.71, 95% CI 1.51–4.86), and pre-procedural TIMI flow grade = 0 (aOR = 2.69, 95% CI 1.43–5.04) after adjusting for relevant clinical variables. Importantly, during a median follow-up of 1.7 years STEMI patients with SIH with or without diabetes experienced increased occurrence of MACCE both in univariate (HR = 1.92, 95% CI 1.19–3.01) and multivariate analysis (aHR = 1.802, 95% CI 1.01–3.21).

**Conclusions:**

SIH in STEMI patients with or without diabetes was independently associated with increased MACCE. This could be attributed to the fact that SIH was strongly correlated with poor pre-procedural TIMI flow, LTB, and distal embolization. Large clinical trials need to validate SIH as an independent predictor of adverse angiographic and clinical outcomes to provide optimal individualized care for patients with STEMI.

**Supplementary Information:**

The online version contains supplementary material available at 10.1186/s12933-022-01578-6.

## Introduction

Stress induced hyperglycemia (SIH) constitutes a transient elevation of blood glucose in the setting of acute illness, including ST segment elevation myocardial infarction (STEMI) [1]. The American Heart Association (AHA) Scientific Statement on Hyperglycemia and Acute Coronary Syndrome recommended that SIH can be diagnosed in patients presenting with STEMI and blood glucose on admission > 140 mg/dl [2].

SIH is associated with increased short and long term major adverse cardiovascular events in patients with STEMI irrespective of the presence of diabetes [3–5]. However, the metabolic milieu in which SIH emerges during epicardial coronary artery thrombosis remains poorly understood. Emerging evidence suggests that acute elevation of blood glucose levels during stress is caused primarily due to acute insulin resistance and upregulation of the metabolic pathways of gluconeogenesis and glycogenolysis [6]. This neuroendocrine dysregulation hampers mitochondrial function and leads to an excessive oxidative stress response. Through this complex pathophysiology, SIH is correlated with a pro-coagulative state, vascular inflammation, and adverse cardiac remodeling [7–10].

Hence, since increased oxidative stress and inflammation during SIH could theoretically produce poor angiographic outcomes, potentially related to increased thrombus burden (TB), distal embolization, and presence of residual thrombus after primary percutaneous coronary intervention (PPCI), an individualized approach for the treatment of this specific high-risk sub-group of STEMI patients is necessary. Yet, intensive insulin therapy studies have failed to show a difference in mortality between insulin-treated and non-insulin treated patients with SIH and acute myocardial infarction [11, 12]. A recent study reported that thrombus aspiration (TA), although not routinely recommended by current ESC guidelines might be beneficial in this subgroup of STEMI patients, as it was associated with lower mortality [13, 14].

To date, only a few studies have evaluated the association between stress hyperglycemia and angiographic predictors of adverse clinical outcomes [15, 16]. The aim of this study was to assess the association of SIH with major adverse cardiovascular and cerebrovascular events (MACCE) and evaluate the effects of SIH on angiographic findings known to be linked with worse prognosis, such as Thrombolysis In Myocardial Infarction (TIMI) flow prior to PPCI, intracoronary TB, distal embolization, and presence of residual thrombus after PPCI in STEMI patients.

## Methods

### Study design

This post-hoc analysis of the prospective CorLipid Trial (NCT04580173) aimed to investigate the association of stress hyperglycemia with adverse angiographic and clinical outcomes of patients with STEMI. The study protocol has been approved by the Scientific Committee of AHEPA University Hospital (Protocol number 321/2019) and the detailed protocol has been previously described [17]. Every trial procedure was conducted according to the principles set by the declaration of Helsinki. Each participant provided written informed consent before being enrolled in the study.

### Eligibility criteria

This study included consecutively enrolled patients with STEMI undergoing coronary angiography in a tertiary academic hospital during the time range of July 2019 to May 2021. Exclusion criteria of the study were: (i) cardiopulmonary arrest upon admission, (ii) patient fasted < 8 h (iii) missing glycemic values on admission, (iv) late presentation of STEMI (> 12 h of pain onset) or thrombolysis.

### Data sources

Angiographic and laboratory characteristics were recorded for every study participant. All blood samples were collected prior to coronary angiography and were analyzed immediately in the in-house clinical laboratory with standard assays.

### Definitions

The diagnosis of STEMI was based on clinical symptoms of myocardial ischemia, electrocardiographic findings consistent with STEMI, and increased high sensitivity troponin values, according to the criteria of the Fourth Universal Definition of Myocardial Infarction [18]. SIH was defined as admission glucose values (AGV) greater than 140 mg/dl (7.8 mmol/L) as defined by AHA [2]. Patients with DM were defined by: (a) administration of any anti-diabetic medication; or (b) diagnostic code of DM (International Classification of Diseases-10) in at least one hospital admission or any previous physician-assigned DM diagnosis; or (c) HbA1c value measured during hospitalization above 6.5% (48 mmol/mol).

### Coronary angiography and angiographic outcomes

Prior to performing PPCI, each patient received guideline-directed pharmaceutical therapy, including unfractionated heparin (80 IU/kg) and a loading dose of aspirin (325 mg). The choice of P2Y12 inhibitor, ticagrelor (180 mg), prasugrel (60 mg) or clopidogrel (600 mg) was at the discretion of the treating physician [19]. Coronary angiograms were analyzed by two experienced interventional cardiologists in a blinded manner. Percentage of diameter stenosis and lesion length were also calculated using quantitative coronary angiography. Patients with pre-procedural TIMI Flow grade 0 were considered to have poor initial TIMI Flow. Angiographic TB was evaluated according to the modified TIMI thrombus classification scale [20]. Based on this classification, patients with TIMI Grade 5 thrombus were reclassified to another thrombus category (G0–G4) after flow achievement with either guidewire crossing or a small non-inflated balloon. Patients with TIMI grades G0–G3 were considered as patients with small thrombus burden (STB), whereas patients with thrombus grade G4 were defined to have large thrombus burden (LTB) [21]. Furthermore, post-procedural antegrade coronary flow was evaluated based on TIMI classification. A patient was considered to have angiographically evident residual thrombus, if modified TIMI thrombus grades G2–G4 were present post-procedurally [22]. The presence of distal embolization or myocardial no-reflow phenomenon were also recorded [23].

### Clinical outcomes definition and follow-up data

The clinical endpoint was a composite of any major adverse cardiovascular and cerebrovascular event (MACCE), defined as the composite of cardiovascular death, stroke, major bleeding, and CVD-related hospitalizations. If a patient suffered several MACCEs during study follow-up, only one was counted in the calculation of MACCE occurrence. Follow-up data on clinical outcomes were collected by trained independent investigators through telephonic or in-person interviews with the study participants. The vital status of patients was also verified by telephonic follow-up or by accessing the Greek Civil Registration System.

### Statistical analysis

Continuous variables are presented as means with standard deviations (SD) and compared using the Mann Whitney U test. Categorical variables are presented as frequencies with percentages and compared via the Pearson chi-square test. Univariable logistic regression analyses were carried out to clarify the association of demographic and clinical baseline characteristics including SIH with angiographic outcomes. The variables with p value < 0.1 were later inserted into multivariable regression models to identify independent predictors of adverse angiographic outcomes. Stratified bootstrapping was used to account for the non-parametric nature of the data.

Kaplan Meier survival curves were developed to investigate the association of SIH with MACCE occurrence. Univariable Cox regression analyses were performed to identify significant predictors of MACCE occurrence among the clinically relevant parameters: SIH, baseline clinical, demographic, and angiographic characteristics. The multivariable Cox regression hazard model was developed by forcing into the multivariable analysis clinically relevant baseline covariates and variables that were univariably significantly associated with MACCE occurrence [age, gender, body mass index (BMI), smoking history, hypertension, DM, dyslipidemia, chronic kidney disease (CKD) and peripheral vascular disease]. The generated adjusted hazard ratios (aHR) are presented along with their respective 95% confidence intervals (CI). A two-sided p-value of less than 0.05 was considered statistically significant. All analyses were conducted through the SPSS v26 (SPSS software, Chicago, IL, USA).

## Results

From July 2019 to May 2021 a total of 653 patients with STEMI were screened for eligibility. Of those, 309 patients (248 males, 61 females) fulfilled the inclusion criteria (Study Flow Diagram in Additional file 1: Fig. S1) and were included in the analysis. Specifically, 121 STEMI patients (39.2%) had AGV more than 140 mg/dl, and a fasting period of at least 8 h. These patients were diagnosed with SIH. The baseline clinical and demographic characteristics of our population are detailed in Table 1. The mean age of the patients was 60.4 ± 12.0 years, and 80.3% were male. Of the baseline demographic and clinical characteristics detailed in Table 1, age, gender, BMI, DM, dyslipidemia, and CKD prevalence differed significantly among patients with and without SIH, while mean glucose, creatinine and triglyceride levels were higher in patients with SIH (p values < 0.05).

**Table 1:** Baseline clinical and demographic characteristics by the presence of stress induced hyperglycemia

	Without SIH	SIH	P-value
	N (%)	N (%)	
Male gender	160 (85.10%)	88 (72.70%)	**0.008**
Hypertension	66 (35.10%)	54 (44.60%)	0.094
DM	10 (5.30%)	47 (38.80%)	**< 0.001**
Dyslipidemia	37 (19.70%)	36 (29.80%)	**0.042**
CAD Family History	47 (25%)	26 (21.50%)	0.479
Smoking	125 (66.50%)	78 (64.50%)	0.715
Previous Stroke	3 (1.60%)	6 (5%)	0.087
CKD	2 (1.10%)	6 (5%)	**0.036**
PAD	5 (2.70%)	3 (2.50%)	0.092
LM	3 (1.6%)	4 (3.3%)	0.325
LAD	126 (67%)	78(64.5%)	0.644
LCx	77 (41%)	47 (38.8%)	0.712
RCA	115 (61.2%)	81 (66.9%)	0.305

	Mean ± SD	Mean ± SD	P-value
Age (years)	58 ± 11	64 ± 13	**0.001**
BMI (kg/m^2^)	29 ± 11	29 ± 4	**0.026**
Glucose (mg/dl)	104 ± 17	201 ± 65	**0.001**
Creatinine (mg/dl)	0.95 ± 0.32	1.14 ± 0.64	**0.007**
Cholesterol (mg/dl)	168 ± 41	161 ± 49	0.081
Triglyceride (mg/dl)	134 ± 63	167 ± 209	**0.035**
TnT-hs (U/L)	1937 ± 2396	2462 ± 3629	0.424
LVEF (%)	45 ± 8	43 ± 10	**0.018**

DM: diabetes mellitus; CAD: coronary artery disease; CKD: chronic kidney disease; PAD: peripheral artery disease; LM: left main artery; LAD: left anterior descending artery; LCx: left circumflex artery; RCA: right coronary artery; BMI: body mass index; LVEF: left ventricular ejection fraction; Statistically significant results are marked in bold

### Stress hyperglycemia and angiographic outcomes

Analysis of the angiographic outcomes according to the presence or not of SIH showed that pre- and post-procedural TIMI flow was significantly lower in patients with SIH (Table 2). LTB and distal embolization occurred more frequently in patients with SIH.

**Table 2:** Angiographic outcomes in STEMI patients with and without stress induced hyperglycemia

	Without SIH	SIH	OR (95% CI) Univariate analysis
	N (%)	N (%)	
Pre-procedural TIMI flow < 1	88 (45.80%)	81 (66.90%)	**2.69 (1.43–5.04)**
Myocardial no reflow	20 (10.60%)	21 (17.40%)	1.76 (0.91–3.42)
Distal embolization	34 (18.10%)	50 (41.30%)	**3.19 (1.90–5.36)**
LTB	67 (35.60%)	68 (56.20%)	**2.19 (1.37–3.49)**
Residual thrombus	20 (10.60%)	12 (9.90%)	0.93 (0.44–1.97)
Poor post-procedural TIMI flow ≤ 2	24 (12.7%)	25(20.8%)	1.72 (0.85–3.45)

LTB: Large thrombus burden, Statistically significant results are marked in bold

Univariate logistic regression analyses showed that patients with SIH were more likely to have distal embolization, LTB and pre-procedural TIMI flow = 0 (Table 2). In bootstrapped multivariable logistic regression models adjusted for age, gender, Hypertension, DM, dyslipidemia, CKD, smoking, Peripheral Vascular Disease and BMI, SIH was an independent predictor of distal embolization (aOR = 2.71, 95% CI 1.51–4.86), LTB (aOR = 2.17, 95% CI 1.27–3.71), and pre-procedural TIMI flow = 0 (aOR = 2.69, 95% CI 1.43–5.04) irrespective of the presence of DM (Table 3). SIH did not affect the occurrence of angiographically evident residual thrombus, no-reflow phenomenon or worse post-procedural TIMI flow.

**Table 3:** Multivariable analysis for the occurrence of: A. Distal embolization, B. Large thrombus burden, C. Poor pre-procedural TIMI flow

	Distal embolization			Large thrombus burden			Pre-procedural TIMI flow < 1		
	aOR	95% CI	P-value	aOR	95% CI	P-value	aOR	95% CI	P-value
Age	1.022	(0.997–1.048)	0.075	1.017	(0.994–1.040)	0.141	1.012	(0.990–1.035)	0.300
Male gender	1.435	(0.742–2.778)	0.275	1.483	(0.795–2.765)	0.216	1.130	(0.606–2.107)	0.700
Hypertension	1.337	(0.731–2.445)	0.343	0.901	(0.519–1.565)	0.711	1.457	(0.844–2.513)	0.176
DM	1.098	(0.545–2.211)	0.801	0.875	(0.439–1.743)	0.703	1.710	(0.841–3.477)	0.139
Dyslipidemia	1.008	(0.535–1.901)	0.470	0.805	(0.448–1.448)	0.470	0.623	(0.345–1.125)	0.116
Smoking	1.200	(0.659–2.186)	0.554	1.751	(1.022–3.001)	**0.041**	1.139	(0.669–1.939)	0.630
CKD	0.315	(0.054–1.841)	0.144	0.224	(0.039–1.285)	0.093	2.393	(0.504–11.369)	0.272
PAD	0.854	(0.158–4.622)	0.731	1.348	(0.317–5.729)	0.686	2.006	(0.439–9.159)	0.369
BMI	1.020	(0.987–1.053)	0.120	1.031	(0.985–1.080)	0.191	0.988	(0.957–1.019)	0.447
SIH	2.706	(1.506–4.863)	**0.002**	2.171	(1.270–3.709)	**0.005**	2.690	**(1.435–5.041)**	**< 0.001**

DM: diabetes mellitus; CKD: chronic kidney disease; PAD: peripheral arterial disease; BMI: body mass index; SIH: stress induced hyperglycemia

Each column represents a different multivariable regression model for each dependent predictor (1: Distal embolization, 2: Large thrombus burden, and 3: Pre-procedural TIMI flow < 1. Each row represents a different independent predictor Statistically significant results are marked in bold

### Survival analysis

**Fig. 1:**
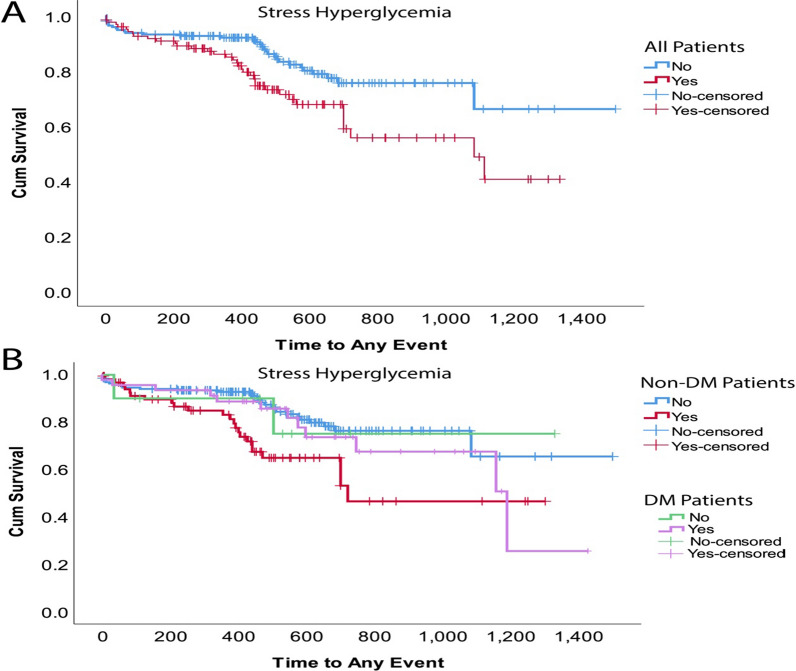
Kaplan Meier survival curve for the occurrence of any major adverse cardiovascular event according to the presence of stress induced hyperglycemia with or without diabetes

As displayed in Fig. 1, SΙH was significantly associated with higher risk of MACCE occurrence during a median follow-up of 1.7 years (Log-rank p < 0.01). Cox regression analysis identified SΙH as a significant predictor of MACCE univariably (HR = 1.92, 95% CI 1.19–3.01) and after multivariable adjustment (adjusted HR = 1.802, 95% CI 1.01–3.21). The multivariable hazard model was adjusted for: age, gender, BMI, heart rate, smoking, DM, dyslipidemia, CKD, number of diseased arteries, distal embolization, LTB, angiographically evident residual thrombus, and post-procedural TIMI Flow. Table 4 summarizes the occurrence of each individual outcome according to the presence of SΙH.

**Table 4:** Follow-up outcomes by the presence of stress induced hyperglycemia

	Without SIH	SIH	P value*
	N (%)	N (%)	
Stroke	0 (0%)	2 (1.70%)	**0.032**
Cardiac hospitalisation	7 (3.70%)	4 (3.30%)	0.660
Bleeding	5 (2.70%)	4 (2.50%)	0.340
Death	22 (11.70%)	31 (25.60%)	**0.002**
MACCE	32 (17%)	37 (30.60%)	**0.005**

## Discussion

This is the first study to investigate the association between SIH and multiple angiographic predictors of poor clinical outcomes in patients with STEMI undergoing PPCI. In addition, this study demonstrates that SIH is significantly associated with worse long-term prognosis irrespective of the presence of diabetes.

Mortality and stroke rates were higher in SIH patients, while cardiac hospitalization and major bleeding events did not differ significantly between patients with and without SIH. A previous study reported increased incidence of cardiac hospitalization among non-diabetic patients with STEMI and SIH [24]. However, a different definition of SIH was used in that study.

Emerging evidence outlines the strong and independent association of SIH with short- and long-term clinical outcomes in STEMI patients irrespective of the definition used for the diagnosis of SIH [3, 5, 25, 26]. However, the exact mechanisms of this correlation are not fully understood. Considering that SIH is linked with adverse angiographic outcomes based on an appealing biological hypothesis as mentioned above, the worse clinical outcomes in SIH patients could, in part, be explained by the higher incidence of angiographic predictors of adverse clinical events before PPCI.

Indeed, our study revealed that patients with STEMI and SIH with or without diabetes were almost three times more likely to have poor pre-procedural TIMI flow. This finding is in line with other findings from previous studies that reported the strong association between SIH and poor-pre procedural TIMI flow [27]. Of note, DM was not associated with poor pre-procedural TIMI flow, even though STEMI patients with DM have worse clinical outcomes [28, 29]. This finding could possibly indicate that SIH might be a major driver of augmented prothrombotic and inflammatory burden during STEMI even in patients with chronic DM.

In this study, almost 6 out of 10 patients with STEMI and concomitant SIH had LTB. It must be emphasized that SIH with or without DM was an independent predictor of LTB after adjusting for clinical risk factors strongly linked with higher intracoronary thrombus burden. Other studies have also verified the strong correlation between SIH and LTB in patients presenting with STEMI [16, 30]. These findings could be explained by the fact that thrombus formation and SIH share common pathophysiology. Specifically, during STEMI oxidative stress from metabolic derangements, including stress hyperglycemia, leads to endothelial injury. Endothelial dysfunction causes decreased nitric oxide availability and disruption of the endothelial cell lining [31]. All these mechanisms give rise to increased adhesion of inflammatory molecules, prothrombotic factors and platelet activation intensifying the formation of thrombus [32]. However, whether SIH is causally related to increased thrombogenicity in patients with STEMI remains to be clarified in future studies.

Regarding other angiographic findings, SIH was significantly associated with more frequent distal embolization. This could be attributed to the fact that distal embolization occurs more often in the presence of LTB during PPCI [33].

The existence of residual thrombus and poor post-procedural TIMI flow did not differ between STEMI patients with and without SIH. The significance of the reported findings is highlighted by the fact that poor procedural TIMI flow during PPCI, LTB and distal embolization are all determinants of larger infract size, lower left ventricular ejection fraction and MACCE [34–36].

Therefore, early identification of those high-risk patients before PPCI is important, as they might benefit from more aggressive therapies, such as use of IIb/IIIa inhibitors and thrombus aspiration. Indeed, a recent study provided evidence of a mortality benefit among STEMI patients with SIH who underwent TA during PPCI, suggesting that the mere quantitative evaluation of thrombus burden cannot adequately identify patient subgroups, who might benefit from mechanical thrombus removal [13].

### Study limitations

Our study has several limitations. Firstly, this was a non-randomized observational cohort study, and all patients were enrolled in a single center, suggesting that potential unknown confounders and selection bias could have influenced the results. Secondly, the diagnosis of SIH was established based on the AHA criteria and all patients reported fasting for at least 8 h before inclusion. However, recent evidence suggests that stress induced hyperglycemia ratio (SIHR) could be a better indicator of stress hyperglycemia especially in diabetic patients. Still, HbA1C% is not always available in the time constraint setting of STEMI, and therefore the diagnosis of SIH based on the definition of SIHR before PPCI is not always feasible. Moreover, intracoronary imaging was not performed. Hence, the presence of intracoronary residual thrombus was based on visual examination of the coronary angiograms.

In conclusion, SIH in STEMI patients undergoing PPCI appears to be associated with poor pre-procedural TIMI flow, LTB, and distal embolization and linked to increased incidence of MACCE. Although the hyperglycemic milieu seems to play a pivotal role in the regulation of oxidative stress and the prothrombotic and inflammatory burden in the STEMI setting, SIH is not currently perceived as an independent risk enhancer in patients with STEMI. Investigating SIH as an additional risk factor in traditional risk scores could facilitate risk-stratification and potentially suggest novel therapeutic targets, promoting personalized care in STEMI patients. Larger and prospective studies are warranted to confirm these proof-of-concept findings.

## Supplementary Information

**Additional file 1**: Figure S1. Flow-diagram.

## Data Availability

Data and study materials are available upon request.
