# Supplementary figures and images for: Association of stress induced hyperglycemia with angiographic findings and clinical outcomes in patients with ST-elevation myocardial infarction
Source: Cardiovasc Diabetol. 2022 Jul 26;21:140. doi: 10.1186/s12933-022-01578-6 (PMC9327277; doi:10.1186/s12933-022-01578-6)

**Supplementary Figure 1. Flow- Diagram**

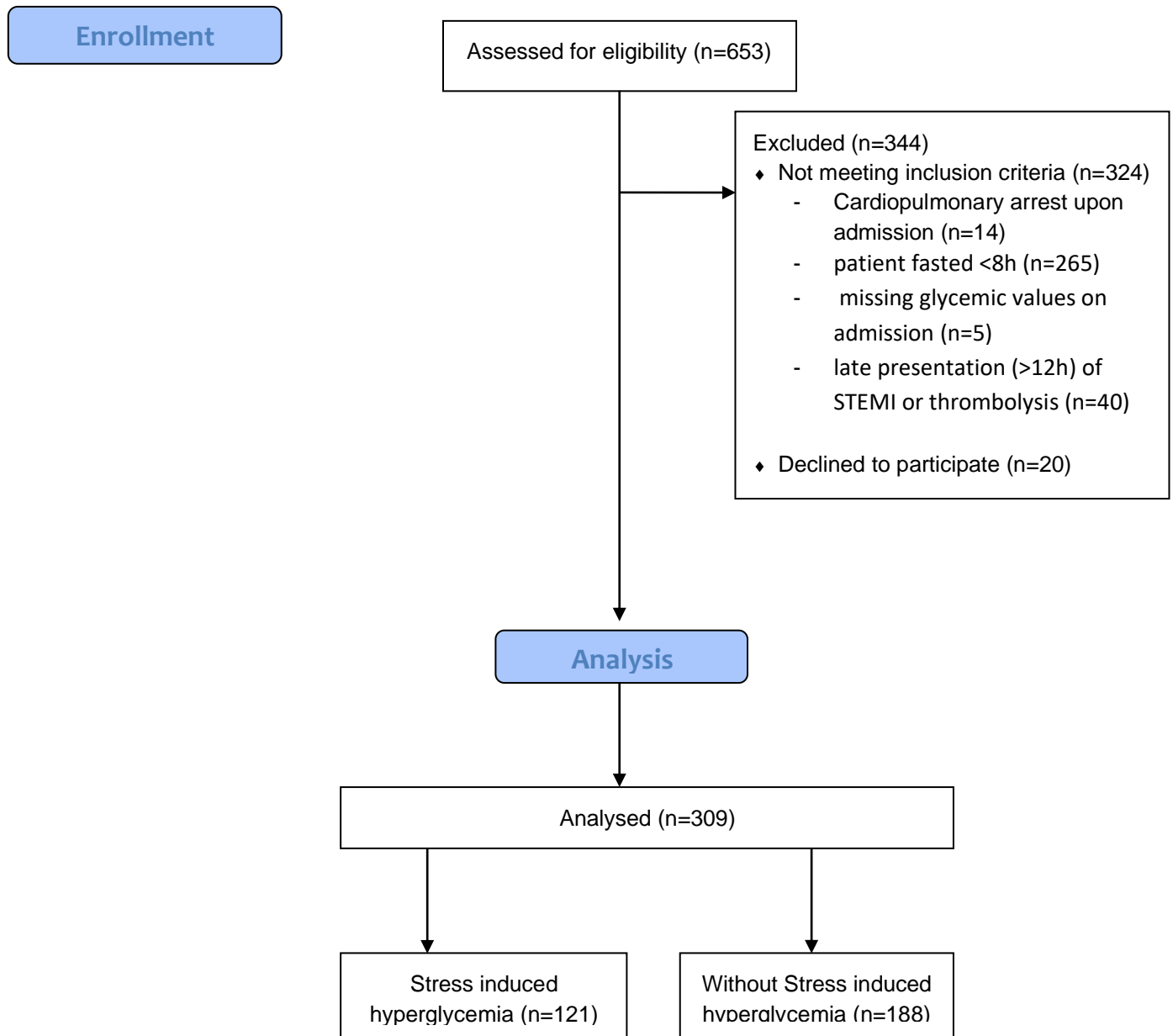

Supplement: Supplementary file 1 — Additional file 1: Figure S1. Flow-diagram. [file 12933_2022_1578_MOESM1_ESM.pdf]
